# Retrotransposon Activates Ectopic *Ptf1* Expression: A Short Tail

**DOI:** 10.1371/journal.pgen.1003331

**Published:** 2013-02-21

**Authors:** Bruce A. Hamilton

**Affiliations:** Institute for Genomic Medicine, Department of Cellular and Molecular Medicine, Department of Medicine, and Moores UCSD Cancer Center, University of California San Diego, La Jolla, California, United States of America; The Jackson Laboratory, United States of America

**Figure 1:**
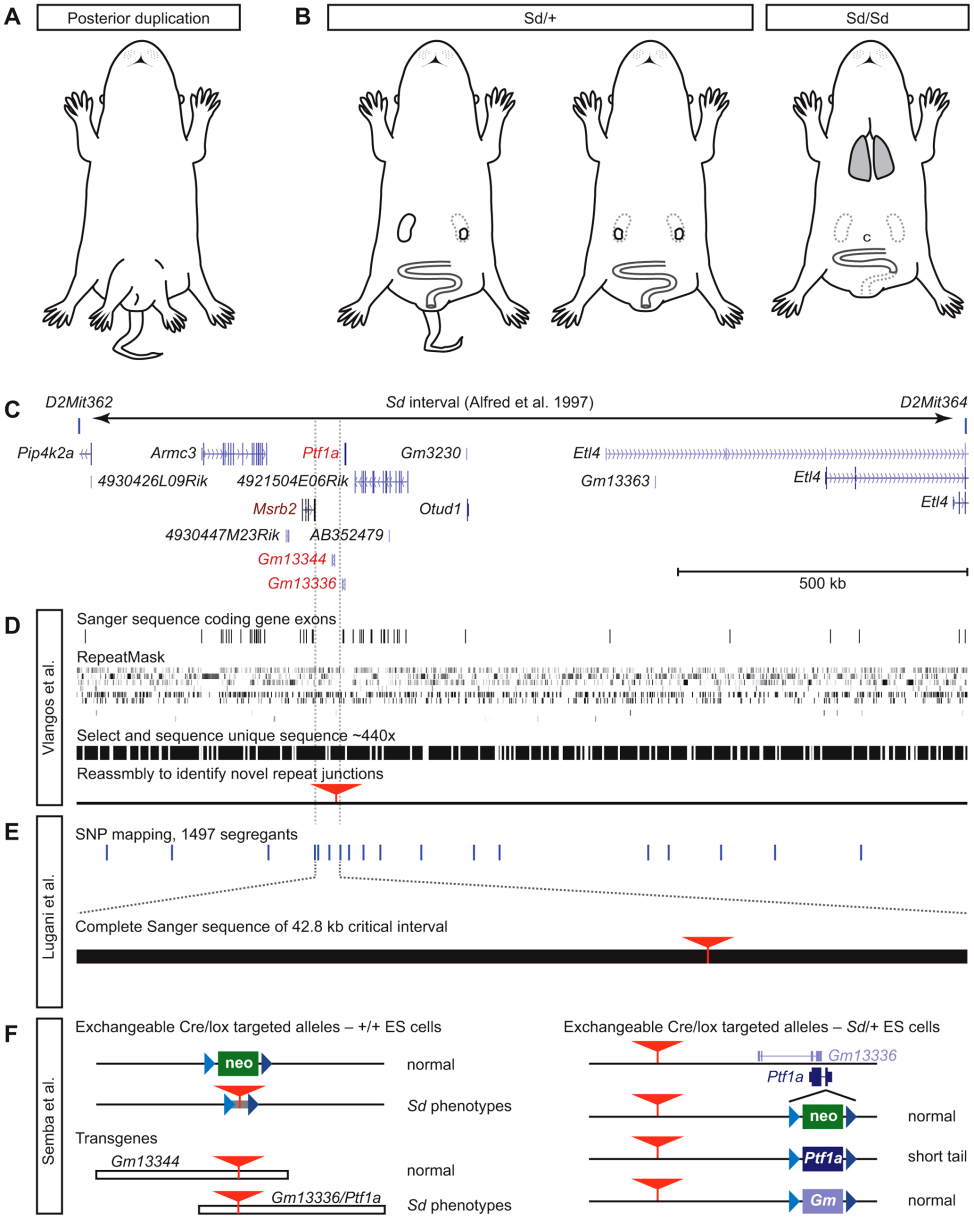
The tale of *Danforth's short tail*. (A) Mice from Danforth's original stock showed posterior duplications, including duplicated hind limbs and a pelvic bulge, in addition to kinked and sometimes shortened tails. Drawing idealized from photographs in [2]. (B) *Sd*/+ mice show a strain-dependent range of caudal phenotypes, including kinked, shortened, or absent tail and reduction or loss of one kidney, but without pattern duplications. *Sd*/*Sd* animals die at birth with caudal regression, including malformation of vertebrae, absent tail, loss of both kidneys, persistent cloaca (c), aganglionosis of the hindgut, and absence of an anal opening. Semba et al. [7] also show that lungs have not inflated. (C) The *Sd* interval of Alfred et al. [11], re-interpreted with gene annotation from the UCSC Genome Browser, shows parts of seven protein-coding genes and seven non-coding transcripts as positional candidate genes. (D) Vlangos et al. [5] report no mutations in exons (horizontal lines), nor in unique sequences obtained by oligonucleotide selection of RepeatMasked genomic sequence (black bars). Reassembly to identify unique sequences whose paired-end reads might identify repetitive sequences not present in the reference genome identifies a novel ETn insertion (red triangle) in an intergenic region 5′ to *Ptf1a*. (E) Lugani et al. [6] report high-resolution recombination mapping with known SNPs (horizontal lines) to limit the *Sd* critical region to 42.8 kb. Exploded view shows the completely sequenced region, the ETn (red triangle) as the only variant in *Sd*. (F) Semba et al. [7] demonstrate both the functional significance of the ETn and the requirement for a Ptf1a open reading frame using serial gene targeting of wild type–and *Sd*/+-derived ES cells and conventional transgenic mice. Blue triangles, variant loxP sites; red triangles, ETn insertion; colored boxes, *neo* (green), *Ptf1a* (deep purple), and *Gm13336* (light purple) replacement cassettes.

In 1925, Charles Danforth wrote about “mice with six legs [that] appeared about two years ago in a stock which had descended from five individuals and had been inbred for several generations” [1]. Danforth studied this “*duplicitas posterior*” (including duplication of internal and external urogenital organs, with quadrilateral symmetry) and its genetic transmission for several years [2], but over time he derived a line in which the principal characteristic was dominant transmission of a short, kinky, or absent tail, eponymously described as the *Danforth's short tail* (*Sd*) mutation [3] (Figure 1A and 1B). Internal caudal regression phenotypes are more serious and include defects in the axial skeleton caused by early degeneration of the notochord; small, malformed or absent kidneys; and hindgut abnormalities. Homozygotes die shortly after birth with more severe phenotypes, including complete loss of the tail, loss of both kidneys, lack of innervation along sections of colon, imperforate anus, and persistence of an abnormally small cloaca—a developmentally transient structure in mammals important for urogenital development. Interestingly, analysis of chimeras indicated cell autonomy for defects in the spine and hindgut, but not in kidney [4].

Despite continued interest over several decades, *Sd* resisted efforts to identify the causal gene—until now. In this issue of *PLOS Genetics*, three laboratories independently identify the *Sd* mutation as an 8.5 kb ETn retrotransposon insertion 12.3 kb upstream from the *Ptf1a* gene [5]–[7]. *Ptf1a* encodes a cell type–restricted basic helix-loop-helix transcription factor required for development of the pancreas and cerebellum [8]–[10]. In the new work, all three groups conclude that ectopic expression of *Ptf1* is the causal event in *Sd* mice.

Catherine Keegan and colleagues (Vlangos et al.) took advantage of the published *Sd* map location [11] (Figure 1C) and powerful genomic tools to isolate the mutation [5]. Finding no mutations after Sanger sequencing all the exons of annotated coding genes in the interval, they developed a custom oligonucleotide array to capture all non-repetitive sequences from the interval for massively parallel sequencing, using paired-end reads and requiring both ends to map correctly in the interval to ensure high-quality assembly (Figure 1D). This allowed the group to analyze more than half of the nucleotides in the critical region at a read depth of 440×, but still provided no plausible candidate mutations for *Sd*. Because *de novo* insertions of repetitive sequence are a frequent source of mutations in mice [12], the group reassembled paired-end samples that had been discarded in the first assembly pipeline because one end landed in a repeat. Using this new algorithm, they identified exactly one novel insertion, not present in a reference genome that shares extensive haplotype with the *Sd* chromosome, 12.3 kb proximal to *Ptf1a*. This candidate insertion was not found in any modern strains tested. RT-qPCR experiments showed a striking, dose-dependent increase in *Ptf1a* expression in *Sd*/+ and *Sd*/*Sd* embryos. Genomic and cDNA-based transgenes provide preliminary evidence that broad overexpression of *Ptf1a* causes embryonic lethality, possibly as an extreme example of the severe *Sd*/*Sd* phenotype.

Ali Gharavi and co-workers (Lugani et al.) took a complementary approach, performing SNP-based linkage mapping in 1,497 segregants to refine the *Sd* interval to a remarkably tidy 42.8 kb intergenic region [6] (Figure 1E). Complete Sanger sequencing of this interval by the group yielded a single DNA change relative to reference sequences: the 8.5 kb ETn element. The complete, high-confidence sequencing of the critical region provides a rigorous demonstration that the ETn insertion must be the *Sd* mutation. Both qPCR and *in situ* hybridization assays again confirmed strong ectopic expression of *Ptf1a*, encompassing all *Sd*-affected tissues. No other protein-coding genes in the broader region near the insertion site showed any similar change. A limited analysis of known *Ptf1a* transcriptional target genes failed to identify upregulation induced by the spread of *Ptf1a* in *Sd* embryos, but key Ptf1a targets in ectopic tissues need not be the same as those in its normal sites of expression.

Ken-ichi Yamamura's group (Semba et al.) also began with a conventional positional cloning approach. By physical mapping with an *Sd*/*Sd* cosmid library constructed for that purpose, they found an unexpectedly large fragment containing the 8.5 kb ETn element as a candidate mutation [7]. To test its functional relevance to *Sd*, the group performed a true tour-de-force of mouse genetics, producing a series of targeted and transgenic alleles to determine which gene products were functionally important to the *Sd* phenotype in the context of the ETn (Figure 1F). As the first step of a serial targeting strategy [13], a neomycin resistance cassette flanked by mutant loxP sites was introduced at the same position as the ETn insertion. This did not induce an *Sd*-related phenotype, suggesting that simple disruption of a *cis*-acting sequence is unlikely to explain the defect. However, replacing the *neo* cassette with a fragment containing the *Sd* ETn produced an allele with dosage-sensitive short tail phenotypes, indicating that the insertion of the ETn at this location is sufficient to create an *Sd*-like mouse. However, in addition to *Ptf1a*, this group found overexpression in *Sd* embryos of two adjacent non-coding RNAs (ncRNAs), *Gm13344* and *Gm13336*. A transgene including the ETn, *Ptf1a*, and *Gm13336* (which overlaps *Ptf1a* on the opposite strand) was sufficient to induce caudal phenotypes, but a similar construct containing the ETn and the other ncRNA was not, narrowing the list of functional candidate genes to two. The team then created germline-competent ES cells from *Sd*/+ embryos and serially targeted the *Ptf1a*/*Gm13336* overlap. Integration of a floxed *neo* cassette on the ETn haplotype creates a *Ptf1a* null with the expected pancreatic agenesis, but no tail or other *Sd*-like effects. However, replacing the *neo* cassette with *Ptf1a*, but not *Gm13336*, does phenocopy *Sd*, demonstrating that it is specifically the ETn-dependent expression of *Ptf1a* that triggers the developmental abnormalities that have been studied in *Sd* mice for more than 70 years.

Through what effectors does *Ptf1a* ectopic expression act? Semba et al. provide an initial answer by profiling RNA in both *Sd* and ETn-*Ptf1a* transgenic mice relative to controls. They find down-regulation of *Cdx2*, another key transcription factor, along with three of its known activation targets, *Cyp26a1*, *T*, and *Wnt3a*. While it is not yet clear whether ectopic Ptf1a acts physically at *Cdx2* in ectopic tissues, rather than indirectly through intervening factors, these profiling results provide clues to important pathways whose expression is disrupted as a consequence of *Ptf1a-Sd*. The results from all three groups, along with analyses of target pathways activated or repressed by Ptf1a in target tissues, will now allow us to ask how well, or in which aspects, *Sd* accurately models human caudal malformation and regression syndromes.

Whether the ETn acts on *Ptf1a* by creating a broad enhancer or by blocking an endogenous silencing element remains to be determined. Either answer might provide insight into other ETn-induced regulatory mutations [14], [15] or more broadly for integration of multiple *cis*-regulatory sites in the presence of retroelements. Additional serial targeting constructs that test activity of specific sequences in the ETn or perhaps chromatin conformation capture methods (3C or its more sophisticated derivatives) might help to resolve the details here. In addition, most mutagenic ETn elements are much smaller [16], [17] than that reported here, and the serial targeting strategy could be used to test whether more typical ETn elements confer a similar property to this locus. The discovery of the *Sd* mutation after so many decades might also prompt us to ask how often regulatory mutations might account for the remaining classical alleles that have been refractory to intragenic-centered analysis and exome sequencing.

The unusual nature of the *Sd* mutation also raises a final question: What relationship—if any—does *Sd* have to the original posterior duplication reported by Danforth, for which several specimens included completely duplicated hindlimbs, kidneys, gonads, phalli, and external urogenital openings? Did this stock contain multiple mutations, a more complex retrotransposon-mediated event that resolved into *Sd*, or other mutations unrelated to *Sd*? Perhaps that is another tail.
